# SNAP47 Interacts with ATG14 to Promote VP1 Conjugation and CVB3 Propagation

**DOI:** 10.3390/cells10082141

**Published:** 2021-08-20

**Authors:** Pinhao Xiang, Yasir Mohamud, Honglin Luo

**Affiliations:** 1 Center for Heart Lung Innovation, St. Paul’s Hospital and Department of Pathology and Laboratory Medicine, University of British Columbia, Vancouver, BC V6Z 1Y6, Canada; eric.xiang@hli.ubc.ca (P.X.); yasir.mohamud@hli.ubc.ca (Y.M.); 2 Department of Experimental Medicine, University of British Columbia, Vancouver, BC V6Z 1Y6, Canada; 3 Department of Pathology and Laboratory Medicine, University of British Columbia, Vancouver, BC V6Z 1Y6, Canada

**Keywords:** SNARE, SNAP47, ATG14, autophagy, coxsackievirus B3, VP1

## Abstract

Coxsackievirus B3 (CVB3), an enterovirus (EV) in the family of *Picornaviridae*, is a global human pathogen for which effective antiviral treatments and vaccines are lacking. Previous research demonstrated that EV-D68 downregulated the membrane fusion protein SNAP47 (synaptosome associated protein 47) and SNAP47 promoted EV-D68 replication via regulating autophagy. In the current study, we investigated the interplay between CVB3 and cellular SNAP47 using HEK293T/HeLa cell models. We showed that, upon CVB3 infection, protein levels of SNAP47 decreased independent of the activity of virus-encoded proteinase 3C. We further demonstrated that the depletion of SNAP47 inhibited CVB3 infection, indicating a pro-viral function of SNAP47. Moreover, we found that SNAP47 co-localizes with the autophagy-related protein ATG14 on the cellular membrane fractions together with viral capsid protein VP1, and expression of SNAP47 or ATG14 enhanced VP1 conjugation. Finally, we revealed that disulfide interactions had an important role in strengthening VP1 conjugation. Collectively, our study elucidated a mechanism by which SNAP47 and ATG14 promoted CVB3 propagation through facilitating viral capsid assembly.

## 1. Introduction

Coxsackievirus B3 (CVB3) belonged to the species human *Enterovirus* B in the genus of *Enterovirus* of the family *Picornaviridae* that comprises a group of small, non-enveloped viruses with a positive, single-stranded RNA [1]. CVB3 is a vital study subject for pediatricians because it is one of the most common pathogens associated with myocarditis affecting children and young adults [2]. CVB3 is also known to cause various acute neurological disorders, accounting for 11.6% of aseptic meningitis cases during the 2008 epidemic in China [3,4]. Although CVB3 is one of the leading causes of sudden deaths in infants and children, it has no effective treatment or vaccination. Therefore, viral mechanistic study is still required to explore the optimal solution for CVB3-induced diseases.

Similar to other viruses, CVB3 is obligated to rely on the host cellular machineries to replicate and spread. One of the most widely studied cellular machineries that is subverted by CVB3 is the macroautophagy (or autophagy) pathway [5]. Autophagy is an evolutionarily conserved degradation pathway, which is triggered by various stresses, including nutrient starvation, organelle damage, and foreign pathogen invasion [6,7]. The autophagy initiation complex can drive the formation of a double-membrane vesicle (DMV) called autophagosome around cellular cargos. The mature autophagosome eventually fuses with the lysosome for cargo degradation. There are over 30 autophagy-related (ATG) genes that are vital for autophagy regulation [8]. One of the essential ATGs is ATG14, which is a subunit of the class III phosphatidylinositol 3-kinase complex (PtdIns 3-kinase) critical for autophagosome formation [9]. It is reported that ATG14 is required for directing the PtdIns-3 kinase to the pre-autophagosome structure [10,11]. ATG14 also participates in autophagosome–lysosome fusion, a process driven by autophagic SNARE (soluble *N*-ethylmaleimide-sensitive factor attachment protein receptor) proteins (i.e., syntaxin 17, SNAP29 and VAMP8) [12]. SNARE proteins are a family of membrane-associated proteins that mediate vesicle fusion and intracellular membrane trafficking [13,14]. They are categorized based on their location into two types: vesicle SNAREs (v-SNAREs) located on the vesicle membrane, and target SNAREs (t-SNARES) situated on the target membrane. Interaction between v-SNARE proteins and t-SNARE proteins drives vesicle/membrane fusion. SNARE proteins play an important role in many fundamental cellular processes, including autophagy [8,15].

Autophagy is generally regarded as cytoprotective under viral infection because the autophagy process can selectively degrade viral particles and components (termed virophagy) [7]. However, enteroviruses (EVs), such as CVB3, have evolved to subvert this pathway for pro-viral purposes [5]. It was observed that CVB3 can induce autophagy independent of the canonical initiation complex and subsequently utilize the autophagosome membranes as scaffolds for viral replication [16,17]. CVB3 can also prevent the maturation of the autophagosome by limiting autophagosome–lysosome fusion [18,19], thereby generating very large autophagy-related structures, termed megaphagosomes [20,21]. The mechanism by which CVB3 disrupts autophagosome maturation towards autolysosomes involves the proteolytic processing of SNAP29 by virus-encoded proteinase 3C [18,19].

Structural studies identified a t-SNARE protein, called SNAP47, that closely resembles the structure of SNAP29 [22]. SNAP47 is widely expressed and especially enriched in neuronal tissues, but its physiological function is still largely unclear [22]. Research from the Jackson Laboratory reported that EV-D68 downregulates SNAP47 and that SNAP47 is a host factor required for viral replication and spread [18]. In the current study, we explored the interaction between CVB3 and SNAP47. we showed that protein levels of SNAP47 are reduced upon CVB3 infection and that the knockdown of SNAP47 suppresses viral infection. Moreover, we demonstrated that SNAP47 interfaces with ATG14 to stimulate viral maturation. Our study revealed a new mechanism; that SNAP47 enhances CVB3 propagation.

## 2. Materials and Methods

### 2.1. Cell Culture and Collection

HeLa and HEK293T cells from American Type Culture Collection (ATCC) were cultured in Dulbecco’s Modified Eagle Medium (DMEM) supplemented with 10% fetal bovine serum and 1% penicillin/streptomycin. Cells were harvested with Modified Oncogene Science lysis buffer (MOSLB, 10 mM HEPES with a pH 7.4, 50 mM Na pyrophosphate, 50 mM NaF, 50 mM NaCl, 5 mM EDTA, 5 mM EGTA, 100 µM Na3VO4, 1% Triton X-100) containing protease inhibitors.

### 2.2. Viral Infection and Titer Measurement

HEK293T or HeLa cells (5.5 × 10^5^) were seeded overnight, and then infected with CVB3 at different multiplicities of infection (MOI) at various timepoints as indicated or sham-infected with PBS.

Fifty-percent tissue culture infective dose (TCID50) was used to quantify a viral titer as described [23]. Briefly, HeLa cells (1 × 10^3^) were seeded onto a 60-well Terasaki plate. On the next day, the samples were serially diluted and overlaid on the seeded cells. After 48 h incubation, viral titer was calculated and expressed as plaque-forming unit (PFU)/mL.

### 2.3. Chemical Treatment

HEK293T or HeLa cells (5.5 × 10^5^) were seeded onto a 6-well plate. For starvation experiments, the cells were washed with PBS and then starved in Hank’s Balanced Salt Solution (HBSS) for 6 h to induce autophagy. To block autophagy flux, 200 nM of bafilomycin A1 was added into HBSS. L-Buthionine (S,R)–Sulfoximine (BSO) treatment was performed as previously described [24].

### 2.4. Plasmids and siRNAs

The 3 × Flag-SNAP47 construct was generated by cloning SNAP47 into the multiple cloning site of p3 × Flag-CMV10 vector with KpnI/BamHI enzymes. The primers used to amplify SNAP47 are shown in Table 1. The 3 × Flag-SNAP29 and 3 × Flag-ATG14 plasmids were gifts from Dr. Qing Zhong at the University of Texas Southwestern Medical Center [12]. The scramble and SNAP47 siRNAs were purchased from Santa Cruz Biotechnology. Cells were transfected with plasmids or siRNAs using Lipofectamine 2000 following the manufacturer’s instructions (Thermofisher Scientific, Burnaby, BC, Canada).

### 2.5. In Vitro Cleavage Assay

In vitro cleavage assay was performed as previously described [25]. Briefly, cell lysates overexpressing 3 × Flag-SNAP47 or GFP-SNAP47 were incubated with purified wild-type (WT) or catalytically inactive CVB3 proteinase 3C (0.1 μg) in a cleavage assay buffer (20 mM HEPES pH 7.4, 150 mM potassium acetate, and 1 mM DTT) for the indicated times at 37 °C. Reaction was terminated with 6 × SDS sample buffer, followed by 95 °C denaturation and subsequent Western blot analysis.

### 2.6. Immunoprecipitation

Immunoprecipitation was performed using EZviewTMRed ANTI-FLAG^®^M2 Affinity Gel according to the manufacturer’s instructions (Sigma-Aldrich, Oakville, ON, Canada). In brief, cell lysates overexpressing Flag-tagged proteins were incubated with anti-Flag M2 agarose beads at 4 °C overnight. After three washes, the bound proteins were eluted with 2 × SDS sample buffer and then subjected to Western blot analysis.

### 2.7. Generation of SNAP47-Knockout and ATG14-Knockdown Cells Using CRISPR-Cas9

Following the protocol described by Ran et al. [26], the single guide RNA (sgRNA) targeting SNAP47 (Table 1) was designed and cloned into pSpCas9 (BB)-2A-GFP vector. The plasmids were transformed into DH5α bacteria, isolated via miniprep and subjected to Sanger sequencing to confirm the presence of sgRNA. Verified plasmids were transfected into HEK293T cells using Lipofectamine 2000. Transfected cells underwent fluorescent activated cell sorting using Mo Flo Astrios EQ (Beckman Coulter, Mississauga, ON, Canada) into a 96-well plate at a ratio of one cell per well. Upon reaching confluency, cells were split for clonal validation, and knockout cells were propagated for downstream experiments. Similar transfection approach was used to generate ATG-14 knockdown but without fluorescent-activated cell sorting. 

### 2.8. Cell Fractionation

Virus-infected or WT cells were incubated with hypotonic lysis buffer (20 mMHEPES, pH 7.4, 10 mM KCl with phosphatase and protease inhibitors) for 20 min on ice. Cell lysates were homogenized, followed by a brief centrifugation to remove cell debris. The supernatant was further centrifuged at 55,000 rpm for 1 h to separate the membrane from the cytoplasm fraction.

### 2.9. Western Blot Analysis

The samples were denatured at 95 °C for 5 min in 6 × sodium dodecyl sulfate protein loading buffer (62.5 mM Tris-HCl at the pH of 6.8, 2% (*w*/*v*) SDS, 10% glycerol, 0.01% (*w*/*v*) bromophenol blue, and 1.25 M of dithiothreitol). After denaturation, proteins were separated by sodium dodecyl sulfate-polyacrylamide gel electrophoresis. Following transfer and blocking, membranes were incubated with the primary antibody overnight at 4 °C, and then with the secondary antibody for 1 h at room temperature. The immunoreactive bands were visualized by enhanced chemiluminescence. All antibodies used were in 1:1000 dilution and summarized in Table 1.

### 2.10. Immunofluorescence and Confocal Microscopy

After fixation and permeabilization, cells were blocked for 1 h with 3% bovine serum albumin, followed by incubation with primary antibodies (anti-Flag and anti-VP1) at 4 °C overnight and then secondary antibodies for 1 hr. After washes, coverslips were mounted using Fluoroshield with DAPI. Images were captured with the Zeiss LSM 880 Inverted Confocal Microscopy.

### 2.11. Statistical Analysis

ImageJ was used to quantify the band intensity. Statistical analysis was performed with unpaired Student’s *t*-test or ANOVA variance analysis. *p*-values < 0.05 were considered to be statistically significant. Experiments were repeated at least three times to ensure the consistency of the results.

## 3. Results

### 3.1. CVB3 Infection Perturbs SNAP47 but Not SNAP23 or SNAP25

**Figure 1:**
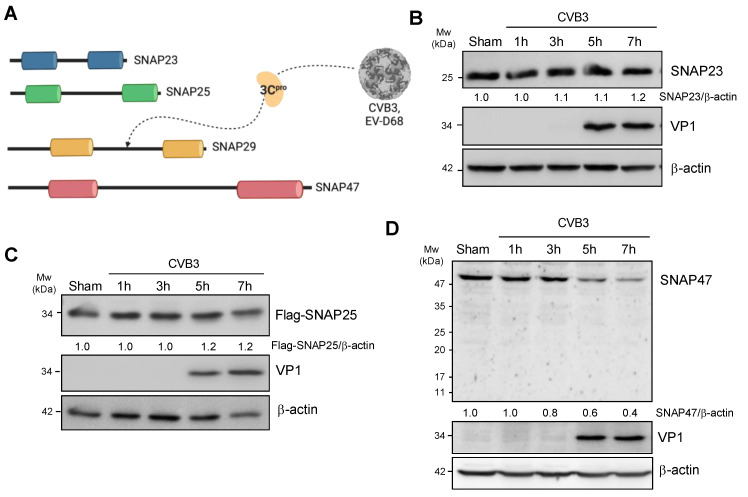
SNAP47 is downregulated following CVB3 infection. (**A**) Schematic diagram illustrating the homologous structure of SNAP23, SNAP25, SNAP29, and SNAP47. Each SNARE carries two homologous SNARE domains. SNAP29 was previously reported to be cleaved by viral proteinase 3C (3C^pro^). (**B**–**D**) HeLa cells (**B**,**D**) or HeLa cells transfected with Flag-SNAP25 (**C**) were infected with CVB3 at an MOI of 10 for indicated timepoints. Western blot analysis was conducted to examine the protein levels of endogenous SNAP23 (**B**), exogenous Flag-SNAP25 (**C**), endogenous SNAP47 (**D**), viral capsid protein VP1, and β-actin (loading control). Densitometry was performed and the ratio of SNAPs over β-actin is presented underneath the blot.

Previous research showed that SNAP29 was an anti-viral protein and cleaved by viral proteinase 3C after CVB3 infection [18,19]. As SNAP23, SNAP25, and SNAP47 all belong to the same SNAP25 family and share structure homology with SNAP29 (Figure 1A, [22]), we hypothesized that SNAP23, SNAP25, and SNAP47 may potentially be involved in CVB3 infection.

To determine the SNARE protein levels after CVB3 infection, HeLa cells or HeLa cells transfected with Flag-SNAP25 (the endogenous level of SNAP25 is extremely low in HeLa cells) were infected with CVB3 at a multiplicity of infection (MOI) of 10 for 1, 3, 5, and 7 h. Cell lysates were collected and subjected to Western blot analysis. As shown in Figure 1B–D, the protein levels of SNAP23 and SNAP25 did not evidently change following CVB3 infection. In contrast, SNAP47 was downregulated, starting at ~5 h post-infection. Viral capsid protein VP1 was blotted as a positive control for CVB3 infection.

### 3.2. Gene-Silencing of SNAP47 Suppresses Viral Infection

**Figure 2:**
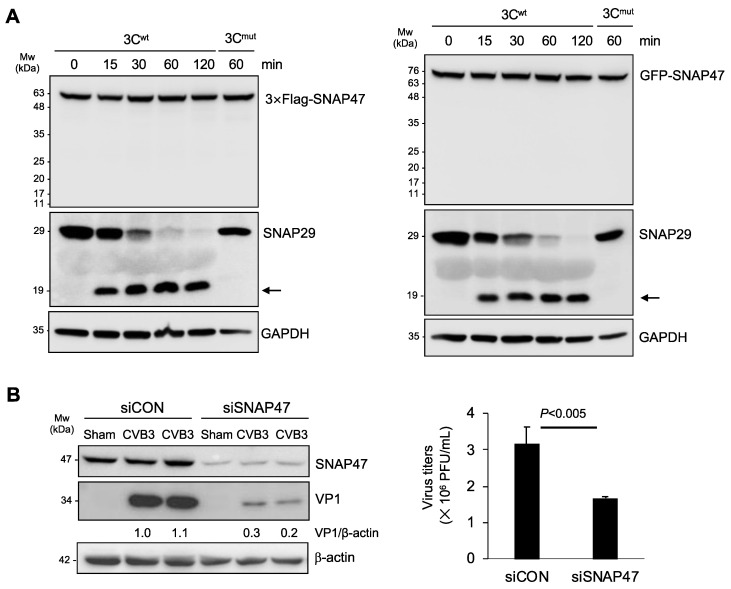
Downregulation of SNAP47 is independent of viral proteinase 3C and results in decreased viral propagation. (**A**) Lysates extracted from HeLa cells overexpressing 3 × Flag-SNAP47 (left panels) or GFP-SNAP47 (right panels) were incubated with purified 3C proteinase for different timepoints for in vitro cleavage assay. 3 × Flag or GFP was blotted to assess the protein levels of exogenous SNAP47. Mutant 3C (3C^mut^) was used as a negative control while SNAP29 was blotted as a positive substrate to confirm proteinase activity. Arrows denote the expected cleavage fragment of SNAP29. (**B**) HeLa cells transfected with control or SNAP47-specific siRNAs were infected with CVB3 at an MOI of 0.1 for 24 h. Western blot analysis was conducted to examine the protein level of VP1 and β-actin (left panel). Densitometry was performed and the ratio of VP1/β-actin is presented underneath the blot. TCID50 assay was conducted to determine viral titer in control and SNAP47-knockdown cells (mean ± SEM, *n* = 3, right).

Next, we explored the potential mechanism and consequence of CVB3-induced SNAP47 downregulation. We and others previously reported that viral proteinase 3C directly cleaves SNAP29 to prevent autophagosome–lysosome fusion [18,19]. As SNAP29 and SNAP47 share the same structure, we speculated that SNAP47 may also be a target of 3C. To test our hypothesis, we carried out in vitro cleavage assays using purified viral proteinase 3C. We incubated 3C proteinase with cell lysates from HeLa cells expressing 3 × Flag-SNAP47 or GFP-SNAP47 for 0, 15, 30, 60, and 120 min. SNAP29 was used as a positive substrate while the catalytically inactive 3C mutant (3C^mut^) served as the negative control for the cleavage assay. In contrast to our original hypothesis, we found that 3C was not responsible for the downregulation of SNAP47 (Figure 2A). The enzymatic activity of the proteinase 3C was confirmed by SNAP29 cleavage. As expected, incubation with 3C^mut^ did not generate cleavage bands for either SNAP47 or SNAP29 (Figure 2A).

We next investigated the role of SNAP47 in viral infection. Following the treatment of HeLa cells with either SNAP47-specific or scramble siRNAs for 48 hours, cells were infected with CVB3 at an MOI of 0.1 for 24 h. Viral infectivity was assessed by the quantification of viral capsid VP1 expression and viral titers. We showed that the knockdown of SNAP47 led to a reduced viral protein expression and titers (Figure 2B), supporting the idea that SNAP47 acts as a pro-viral factor during CVB3 infection.

### 3.3. Ablation of SNAP47 Does Not Impair Starvation- or CVB3-Induced Autophagy

**Figure 3:**
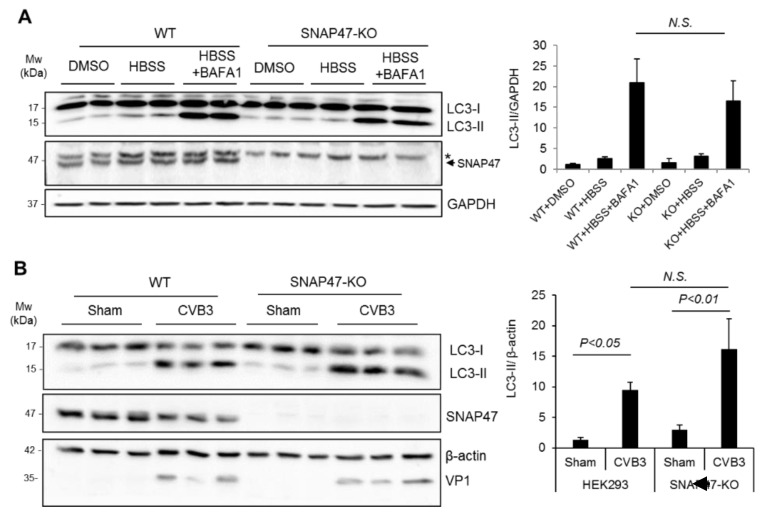
Ablation of SNAP47 does not impair starvation- or CVB3-induced autophagy. (**A**) Wildtype (WT) and SNAP47-knockout (KO) HEK293T cells were starved in HBSS medium in the presence or absence of a lysosomal inhibitor bafilomycin A1 (BAFA1, 200 nM) for 6 h. Western blot analysis was conducted to probe for SNAP47, LC3, and GAPDH. * Indicates non-specific bands. Arrow denotes the location of SNAP47 protein band. (**B**) WT and SNAP47-KO HEK293T cells were infected with CVB3 at an MOI of 100 for 8 h. Western blotting was performed to detect SNAP47, LC3, and β–actin/VP1. LC3-II/GAPDH (**A**) and LC3-II/β–actin (**B**) were quantified by densitometry analysis and are presented as a bar graph (mean ± SEM, *n* = 3). N.S., not significant.

To determine the possible mechanism by which SNAP47 enhances viral growth, we first investigated whether SNAP47 played a role in autophagy. We generated SNAP47-knockout (KO) cell lines in HEK293T cells using the CRISPR-Cas9 gene editing approach. We chose HEK293T cells for this study because of our previous experience; that HEK293T cells have a relatively lower basal autophagy when compared to HeLa cells. WT and SNAP47-KO HEK293T cells were starved in an HBSS medium for 6 hours to stimulate the starvation-induced autophagy. In parallel conditions, cells were treated with an HBSS medium in combination with a bafilomycin A1 (BAFA1, 200 nM), a vacuolar-type H (+) -ATPase inhibitor that prevented lysosome acidification and thereby inhibited autophagosome–lysosome fusion. The successful knockout of SNAP47 was verified by Western blotting (Figure 3A,B). The 50-kDa bands (indicated with *) in Figure 3A were present in all samples, likely due to non-specific antibody binding. The protein levels of LC3-II (15-kDa) were comparable between WT and SNAP47-KO cells after starvation/BAFA1 treatment (Figure 3A), suggesting that autophagy flux was unaffected in SNAP47-depleted cells.

Recent evidence supported the idea that CVB3-induced autophagy bypassed the requirement for canonical autophagy factors, such as the unc-51-like kinase (ULK1) and beclin–phosphatidylinositol 3-kinase (PI3K) complexes, which are crucial for basal and starvation-induced autophagy [16,17]. To test whether SNAP47 functioned as a non-canonical factor participating in CVB3-induced autophagy, we infected SNAP47-KO and WT cells with CVB3. Relative to the sham-infected cells, LC3-II showed an expected increase in both WT and SNAP47-KO cells after 8 hours of viral infection (Figure 3B). However, no statistical difference was observed in LC-II between WT and SNAP47-KO cells following CVB3 infection. VP1 was blotted to confirm the success of CVB3 infection. Collectively, our results suggested that SNAP47 did not appear to be involved in either starvation-mediated canonical autophagy or CVB3-induced non-canonical autophagy.

### 3.4. SNAP47 Interacts with ATG14 on the Cellular Membrane Fractions alongside VP1

**Figure 4:**
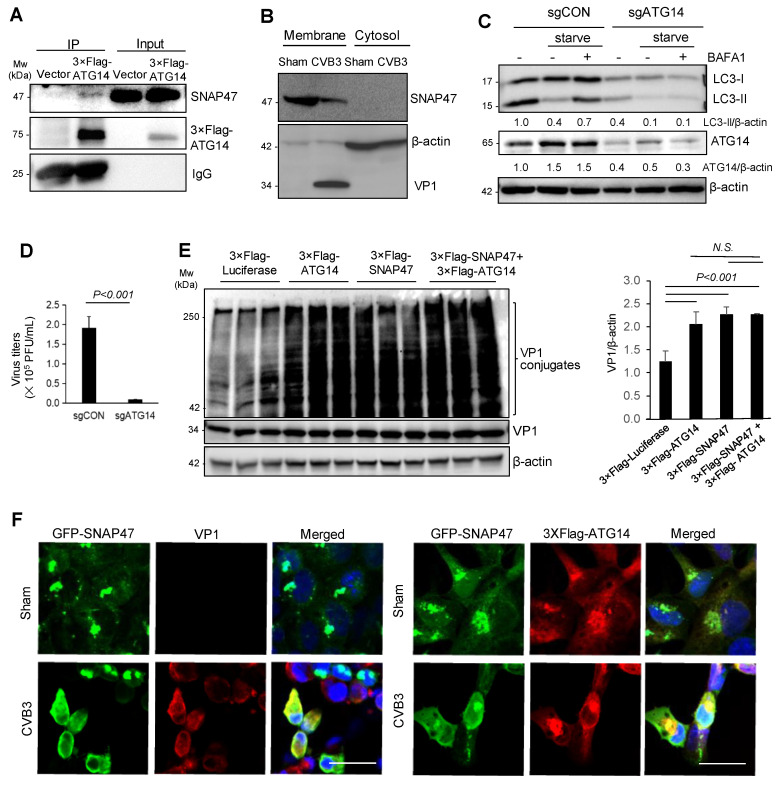
SNAP47 interacts with ATG14 to promote viral capsid assembly on cellular membrane. (**A**) Co-immunoprecipitation was performed on the HeLa cell lysates overexpressing 3 × Flag-ATG14 using anti-Flag antibody. SNAP47 and 3 × Flag-ATG14 were blotted to assess the protein interaction. IgG light-chain was used as a loading control. (**B**) Cell fractionation was performed on the cell lysates harvested from HeLa cells infected with CVB3 or sham-infected (MOI = 10, 7 h) to separate the membrane and cytosol fraction. Western blot was conducted to detect SNAP47, VP1, and β-actin. (**C**) HeLa cells transfected with sgCON (control) or sgATG14 were starved in HBSS medium with or without BAFA1 (200 nM) for 6 h. Western blot was conducted for examination of ATG14, LC3, and β-actin. LC3-II/β-actin and ATG14/β-actin were quantified by densitometry analysis and are shown below the blot. (**D**) HeLa cells depleted of ATG14 with sgRNA were infected with CVB3 (MOI = 0.1) for 24 h. TCID50 was conducted to measure viral titer (mean ± SEM, *n* = 3). (**E**) HeLa cells transfected with 3 × Flag-Luciferase, 3 × Flag-ATG14, 3 × Flag-SNAP47, or 3 × Flag-SNAP47 plus 3 × Flag-ATG14 were infected with CVB3 (MOI = 10) for 7 h. The cell lysates were collected and blotted for VP1and β-actin. T-test was performed on the relative densitometry of VP1 conjugation (mean ± SEM, *n* = 3). (**F**) HEK293T cells transfected with GFP-SNAP47 and 3 × Flag-ATG14 were infected with CVB3 (MOI = 10) for 7 h. Immunocytochemical staining was conducted using anti-Flag and anti-VP1 antibodies. Scale bar = 25 µm.

A previous study reported that SNARE proteins, SNAP29 and syntaxin 17, interacted with the membrane-associated autophagy protein ATG14 to facilitate autophagosome–lysosome fusion [12]. Given the affinity of ATG14 for SNARE proteins, we questioned whether SNAP47 was also physically associated with ATG14. Using a 3 × Flag-tagged ATG14 construct, we identified ATG14 as an interacting partner for SNAP47 by co-immunoprecipitation (Figure 4A). Cell fractionation revealed that SNAP47 and VP1 (a well-known membrane-associated protein) were enriched on the membrane fractions (Figure 4B).

As few studies have examined the role of ATG14 in viral infection, we used the CRISPR-Cas9 gene-editing plasmid expressing a sgRNA targeting the first exon of ATG14 (Table 1) and examined its effects on CVB3 infectivity. Following 48 h of transfection, cells were subjected to sham or CVB3 infection. The CRISPR-based targeting was effective at silencing > 80% of ATG14 expression (Figure 4C). Consistent with ATG14′s role in starvation-induced autophagy, we observed a noticeable decrease in the LC3-I to LC3-II conversion after knockdown of ATG14 (Figure 4C). Compared to control cells, ATG14 ablation resulted in a significant reduction in viral titers (Figure 4D), suggesting a pro-viral role for ATG14, similar to SNAP47.

As both SNAP47 and ATG14 were membrane-associated proteins that co-fractionate with the viral capsid protein VP1, we speculated that their interactions may be involved in viral capsid maturation. To test our hypothesis, we overexpressed constructs expressing ATG14 and SNAP47 separately or in combination and assessed VP1 conjugation as a proxy for viral capsid maturation [1,27]. Figure 4E showed that VP1 conjugates were significantly increased upon the expression of ATG14 and/or SNAP47. Co-expression of SNAP47 and ATG14 did not cause an additive effect, possibly due to the presence of endogenous SNAP47 and ATG14 that masked the role of exogenous proteins when applied together (Figure 4E). We also performed confocal microscopy to visualize the localization of SNAP47, ATG14, and VP1 under sham and CVB3 infection. VP1 staining was not detected in sham-infected conditions as anticipated. In CVB3-infected cells, SNAP47 was re-localized from distinct punctate structures to overlap with ATG14 and VP1 (Figure 4F).

### 3.5. Atg14 Promotes Viral Capsid Maturation via Glutathione-Mediated Disulfide Bond Formation

**Figure 5:**
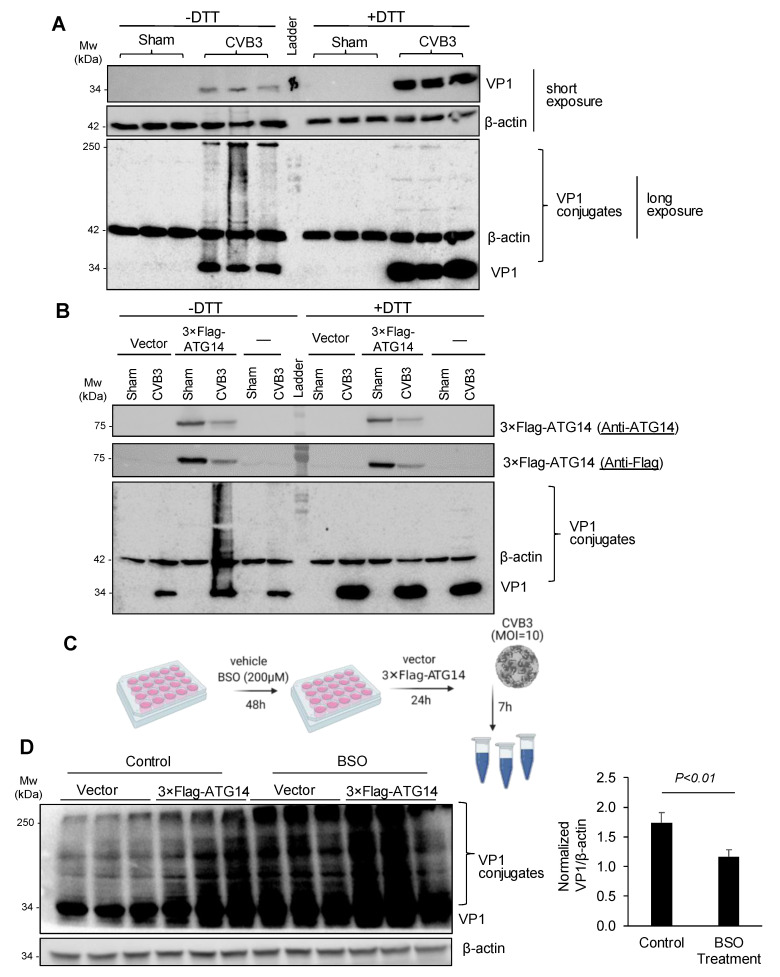
ATG14 promotes glutathione-mediated assembly of VP1. (**A**,**B**) HeLa cells (**A**) or HeLa cells transfected with 3 × Flag-ATG14 (**B**) were sham- or CVB3-infected (MOI = 10, 7 h) in the presence or absence of DTT as indicated. Cell lysates were harvested and Western blot was performed to assess monomeric and conjugated VP1. (**C**) Diagram showing the experimental protocol to test if glutathione was involved in disulfide bridge formation, buthionine sulfoximine (BSO: a glutathione synthesis inhibitor). (**D**) HeLa cells were treated with BSO (200 μM) or a vehicle for 48 h, and then transfected with 3 × Flag-ATG14 or an empty vector for 24 h. Subsequently, the cells were infected with CVB3 (MOI = 10) for 7 h. Cell lysates were collected for Western blot analysis. VP1 conjugates in cells expressing 3 × Flag-ATG14 were first normalized to β-actin, and then to the levels of VP1 conjugates in cells expressing empty vectors in both control and BSO-treated groups (mean ± SEM, *n* = 3).

Previous studies demonstrated that the glutathione-mediated formation of disulfide bonds was required for the viral capsid maturation and effective production of infectious virions [24]. To determine the significance of disulfide interactions in viral capsid maturation, we treated cell lysates with either vehicle or dithiothreitol (DTT), a powerful reducing agent. Figure 5A showed that VP1 conjugates were almost abolished after DTT treatment, accompanied by an increased level of monomeric VP1 at the 34-kDa, suggesting that VP1 conjugates are primarily mediated by disulfide interactions.

We next determined whether ATG14 and SNAP47 promoted viral capsid conjugation through disulfide bridge interactions. Interestingly, ATG14 was previously reported to facilitate self-oligomerization via four proximal cysteine residues that participated in disulfide interactions and SNARE complex formation [12]. We expressed 3 × Flag-ATG14 into HeLa cells with or without CVB3 infection and performed a Western blot on the collected cell lysates in the presence or absence of the reducing agent DTT. We observed that the expression of 3 × Flag-ATG14 significantly enhanced viral capsid conjugation, whereas upon DTT treatment, VP1 conjugation was completely abolished (Figure 5B), confirming that formation of disulfide bonds was critical in driving ATG14-mediated VP1 conjugation.

Lastly, we tested whether glutathione, the major intracellular antioxidant that facilitated redox reactions such as disulfide bridge formation [28], was a host factor for VP1 conjugation. We pre-treated HeLa cells with buthionine sulfoximine (BSO) or a vehicle for 48 h to inhibit glutathione production and then expressed 3 × Flag-ATG14 or an empty vector for 24 h. Subsequently, cells were infected with CVB3 for 7 h and cell lysates were harvested for Western blot analysis (Figure 5C). After normalizing the VP1 conjugates in 3 × Flag-ATG14-expressing cells over empty vector-expressing cells in both control and BSO-treated cells, we showed that ATG14-mediated VP1 conjugation was significantly reduced following BSO treatment although it was not completely abolished (Figure 5D), suggesting that other intracellular antioxidants may also be involved in VP1 conjugation.

## 4. Discussion

The repurposing of host cellular factors is a powerful viral strategy to subvert normal cellular processes in favor of executing the viral agenda. The famous adage, “divide and conquer”, is especially apt when considering the viral modus operandi of hijacking cellular machinery and building viral factories. The proliferation of DMVs that act as viral replication organelles has become a cellular hallmark of active infection for a diverse array of positive-stranded RNA viruses including EVs [29]. Recent evidence supports the idea that EVs initiate the reorganization and remodeling of cellular architecture by usurping non-canonical factors to promote DMV formation [29,30,31]. In doing so, these host factors are repurposed to facilitate viral replication and maturation.

In contrast to previous studies [18], we did not observe a major role for SNAP47 in regulating autophagy. One explanation for such a discrepancy may the different EV strains used (i.e., EV-D68 vs. CVB3). Our results support the idea that SNAP47 is functionally distinct from its close SNARE family protein, SNAP29, in regulating autophagy flux and viral propagation. The gene-silencing of SNAP47 is associated with a significant reduction in viral protein expression and viral titers, but does not appear to affect autophagy flux.

We postulate that SNAP47 controls viral growth through multiple mechanisms at various stages of the viral life cycle. When examining the mechanism behind SNAP47-driven CVB3 replication and spread, we discovered that SNAP47 colocalizes with ATG14 on the cellular membrane fractions together with viral capsid protein VP1, suggesting a functional interaction between SNAP47 and ATG14 may be involved in viral replication. Although the association between ATG14 and SNAREs has been previously reported for SNAP29 and syntaxin 17 [12], it remains unclear whether ATG14 complexes with other SNARE proteins outside the autophagy fusion machinery. Beyond its role in autophagy, ATG14, also known as Beclin-1, associated with the autophagy-related key regulator (Barkor), is a component of the PI3K kinase complex and utilizes its conserved C-terminal Barkor/ATG14 autophagosome-targeting sequence (BATS) domain to anchor to cellular membranes [9,10,12]. Similar to ATG14, our study demonstrated that SNAP47 is predominantly a membrane-associated factor co-fractionating with viral capsid proteins. Given that viral replication organelles are membranous structures that promote viral RNA synthesis, viral assembly and maturation, we reasoned that membrane-associated host factors such as SNAP47 and ATG14 exert their pro-viral functions as a consequence of their close proximity and localization to the replication organelles.

The current study further explored the potential role of SNAP47 and ATG14 in viral capsid maturation. Of note, the expression of SNAP47 and/or ATG14 results in enhanced VP1 conjugation. The observed smearing when probing for VP1 in Western blot analysis supports the stochastic nature of viral capsid assembly that relies on direct and indirect interactions with various host and viral factors. Previous studies implicate glutathione as an essential host factor for viral capsid maturation and virion production [24]. Glutathione is the major non-protein thiol of the cell and utilizes its cysteine moiety as a cofactor in various redox and antioxidation reactions [28,32]. Glutathione also assists in native protein folding in the endoplasmic reticulum of normal cells through its regulation of disulfide bridge formations and tertiary protein structures [32,33,34]. We found that VP1 conjugation mediated by SNAP47 and ATG14 is partially dependent on the availability of glutathione as disruption of the glutathione biosynthetic pathway significantly attenuates VP1 conjugation. Notably, VP1 conjugation is not completely abolished upon the inhibition of the glutathione biosynthetic pathway, suggesting that other cellular mechanisms or host factors may also be involved in VP1 conjugation. One example is α-lipoic acid-driven cellular redox reactions, as α-lipoic acid is often found to covalently attach to multi-enzyme complexes [35]. In addition, evidence from the literature supports the idea that the autophagic membrane protein LC3 (microtubule-associated protein 1A/1B-light chain 3) is among the host factors present at sites of viral replication/maturation and may promote viral conjugation [36].

In this study, it was observed that protein levels of SNAP47 are reduced after CVB3 infection. Despite both SNAP47 and SNAP29 being downregulated following CVB3 infection, SNAP47 was resistant to processing by viral proteinase 3C. The mechanism behind CVB3 subversion and the downregulation of SNAP47 remains an area of active research.

In conclusion, our results suggest that CVB3 actively hijacks host cellular factors, ATG14 and SNAP47, to facilitate viral propagation. One underlying mechanism identified for their pro-viral function is through the enhancement of viral capsid assembly.

## Figures and Tables

**Table 1:** Reagents and Resources.

Chemicals, Peptides, and Recombinant Proteins		
Bafilomycin A1	Sigma-Aldrich	B1793
Buthionine Sulfoximine	Cayman Chemicals	14484
CVB3 recombinant 3^wt^, 3C^mut^	Dr. Eric Jan (University of British Columbia, Vancouver, Canada	Jagdeo et al., 2015
Experimental model: Cell lines		
HeLa	ATCC	CCL-2
HEK-293T	ATCC	CRL-3216
Recombinant DNA		
3×Flag-SNAP29	Dr. Qing Zhong (University of Texas Southwestern Medical Center, TX, USA)	(Diao et al., 2015)
GFP-SNAP47	Current study	
3XFLAG-ATG14	Dr. Qing Zhong (University of Texas Southwestern Medical Center, TX, USA)	(Diao et al., 2015)
Software and Algorithms		
ImageJ	NIH	Version 1.46r
Antibodies		
LC3-I/II (rabbit)	Novus Biologicals, Cat. No. NB100-2220	
GAPDH (rabbit)	Abcam, Cat. No. ab8245	
SNAP 23 (mouse)	Santa Cruz, Cat. No. sc-374215	
SNAP 29 (rabbit)	Abcam, Cat. No. ab181151	
SANAP47 (rabbit)	Abcam, Cat. No. ab172609	
β-Actin (mouse)	Sigma-Aldrich, Cat. No. A5316	
VP1 (mouse)	Dako Cat.No. M706401-1	
Flag (mouse)	Sigma-Aldrich Cat.No. F1804	
DAPI	Sigma-Aldrich, F6057	
Goat-anti-mouse IgG, HRP-linked antibody	Santa Cruz, Cat. No. SC-2005	
Goat-anti-rabbit IgG, HRP-linked antibody	Cell Signaling Technology, Cat. No. 7074	
Oligonucleotides		
SNAP47	Forward 5′AAATTTAGATCTATGCGCGCGGCTCGC 3′ Reverse 5′AAATTTGGTACCCTAGGTCAGCCTCTTCAT CCGCCTGTT 3′	Current study
SNAP47-KO	Forward 5′CACCGAGGCTCACCGTCCTTGTGTC 3′ Reverse 5′AAACGACACAAGGACGGTGAGCCT C 3′	Current study
sgATG14	Forward 5′ CACCGGACTCCGTGGACGATGCGG 3′	Current study

## Data Availability

The data presented in this study are available upon request from the corresponding author.
